# Blunted IL-17 responses early after advent of multiple injuries

**DOI:** 10.1186/cc10616

**Published:** 2012-03-20

**Authors:** M Paraschos, M Patrani, A Pistiki, J Van der Meer, M Netea, E Giamarellos-Bourboulis, K Mandragos

**Affiliations:** 1 Korgialeneion Benakeion Hospital, Athens, Greece; 2 University of Athens, Medical School, Athens, Greece; 3 UMC St Radboud, Nijmegen, the Netherlands

## Introduction

To define the impact of multiple injuries without the presence of sepsis in IL-17 responses.

## Methods

A total of 32 patients and 17 healthy volunteers were enrolled. All patients were bearing: multiple injuries necessitating ICU admission with an injury severity score more than 16; and systemic inflammatory response syndrome. Patients with infections upon ICU admission were excluded from the study. Heparinized venous blood was sampled within the first 24 hours after ICU admission. Peripheral blood mononuclear cells (PBMCs) were isolated after gradient centrifugation of whole blood over Ficoll. They were incubated for 5 days in RPMI 1640 supplemented with 2 mM glutamine and 10% FBS in the presence of 10 ng/ml lipopolysaccharide (LPS) of *Escherichia coli *O55:B5; of 5 μg/ml phytohemmaglutin (PHA); of 5 × 10^5 ^cfu/ml of heat-killed *Candida albicans *(HKCA), of *Pseudomonas aeruginosa *(HKPA) or of *Staphylococcus aureus *(HKSA). IL-17 was measured in supernatants by an enzyme immnunoassay.

## Results

**Figure 1:**
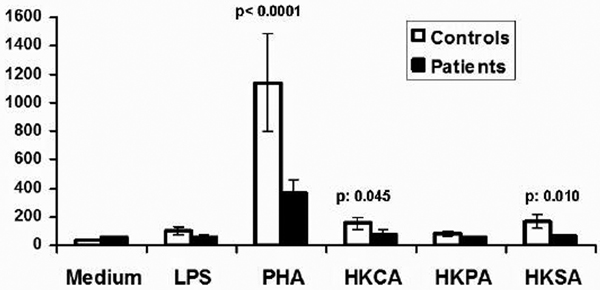
**Release of IL-17 by PBMCs of controls and of patients**.

Mean APACHE II score of patients was 14. Release of IL-17 by PBMCs of patients was significantly lower compared to controls, as shown in Figure 1. *P *values refer to comparisons between controls and patients.

## Conclusion

The presented findings show that early upon advent of multiple injuries IL-17 responses are blunted. This may corroborate with the susceptibility of patients for superinfections.

